# Longitudinal Observation of Changes in the Ankle Alignment and Tibiofibular Relationships in Hereditary Multiple Exostoses

**DOI:** 10.3390/diagnostics10100752

**Published:** 2020-09-25

**Authors:** Jae Hoo Lee, Chasanal Mohan Rathod, Hoon Park, Hye Sun Lee, Isaac Rhee, Hyun Woo Kim

**Affiliations:** 1 Department of Orthopaedic Surgery, Ilsan Paik Hospital, Inje University College of Medicine, Goyang 10380, Korea; jaehoolee@gmail.com; 2 Division of Pediatric Orthopaedic Surgery, SRCC NH Children’s Hospital, Mumbai 400034, India; chasanal@gmail.com; 3 Department of Orthopaedic Surgery, Gangnam Severance Hospital, Yonsei University College of Medicine, Seoul 06273, Korea; hoondeng@yuhs.ac; 4 Biostatistics Collaboration Unit, Yonsei University College of Medicine, Seoul 03722, Korea; hslee1@yuhs.ac; 5 Medical Course, University of Melbourne Melbourne Medical School, Melbourne 3010, Australia; isaac.rhee@gmail.com; 6 Division of Pediatric Orthopaedic Surgery, Severance Children’s Hospital, Yonsei University College of Medicine, Seoul 03722, Korea

**Keywords:** hereditary multiple exostoses, tibia, fibula, ankle, valgus

## Abstract

The longitudinal changes in the tibiofibular relationship as the ankle valgus deformity progresses in patients with hereditary multiple exostoses (HME) are not well-known. We investigated the longitudinal changes and associating factors in the tibiofibular relationship during the growing period. A total of 33 patients (63 legs) with HME underwent two or more standing full-length anteroposterior radiographs. Based on the change in ankle alignments, thirty-five patients with an increase in tibiotalar angle were grouped into group V, and 28 patients with a decreased angle into group N. In terms of the change in radiographic parameters, significant differences were noted in the tibial length, the fibular/tibial ratio, and the proximal and distal epiphyseal gap. However, age, sex, initial ankle alignment, location of osteochondroma, and presence of tibiofibular synostosis did not affect the tibiofibular alignment. The tibial growth was relatively greater than the fibular growth and was accompanied by significant relative fibular shortening in the proximal and distal portions. In pediatric patients with HME, age, sex, initial ankle alignment, location of the osteochondroma, and synostosis did not predict the progression of the ankle valgus deformity. However, when valgus angulation progressed, relative fibular shortening was observed as the tibia grew significantly in comparison to the fibula.

## 1. Introduction

Valgus deformity of the ankle is a common manifestation of hereditary multiple exostoses (HME), affecting approximately half of the patients [1,2,3,4]. It may result in pain, restricted range of motion, and gait disturbances [5]. In a study investigating the natural history of untreated ankle joints in HME patients, 14 out of 75 ankles (19%) with an average tibiotalar tilt of valgus 9° showed early radiographic signs of osteoarthritis [6]. Appropriate surgical correction for excessive tibiotalar tilting has been recommended to preserve ankle function and prevent early arthritic changes in adulthood [7,8,9].

Past studies have suggested that the ankle valgus deformity is caused by a disproportionate shortening of the fibula relative to the tibia [2,4,6,10]. Compared to valgus deformity of the knee, which is primarily caused by changes in the distal femur and proximal tibia, ankle valgus deformity is directly affected by the disparity between the distal tibial and fibular length [1,6]. For this reason, predicting fibular shortening in HME patients may guide the indications for appropriate surgical treatment of ankle valgus.

Characteristic factors that have been associated with higher rates of ankle valgus deformities include an ankle valgus over 10° in males and exostoses on the proximal and distal tibia or fibula [10,11]. However, there is a lack of research analyzing the tibiofibular relationship, especially longitudinal observation studies investigating tibial and fibular growth. Furthermore, the literature is sparse in identifying factors related to the progression of ankle valgus and relative fibular shortening during growth periods.

The purpose of this study was to: (1) analyze longitudinal changes in the tibiofibular relationship of HME patients during their growing period; (2) identify factors associated with the progression of ankle valgus deformities; and (3) determine the effects of predisposing factors on the growth of the fibula and tibia over time.

## 2. Materials and Methods

### 2.1. Patient Recruitment

This retrospective study was approval by the institutional review board of the Severance Hospital, Seoul, Korea (28 November 2013; 4-2013-0700). Medical records and plain radiographs of patients who were skeletally immature at their first visit and underwent two or more full-length standing anteroposterior (AP) radiographs of the lower extremities during their follow-up were reviewed. Patients with a previous surgical history of the lower extremities and a limb-length discrepancy of over 2.5 cm, measured in any of the radiographs, were excluded. Among 53 patients who were initially enrolled, a total of 20 patients were excluded due to surgical history of their lower extremities (16 patients) and limb-length discrepancy of over 2.5 cm (4 patients). A total of 265 full-length radiographs of 63 affected legs in 33 patients were finally included in the study. The cases were divided into two groups of Group V and Group N, according to whether the tibiotalar angle increased into a more valgus position (Group V), or decreased into a more varus position (Group N) at the final evaluation when compared to the initial visit.

### 2.2. Radiographic Measurements

Full-length AP radiographs were taken in the standardized manner with the patients in a bipedal stance with equal weight bearing of both feet [12]. All radiographic parameters were measured using Picture Archiving and Communication Systems (PACS) (Centricity PACS 2.0; GE Medical Systems Information Technologies, Milwaukee, WI, USA). Radiographic parameters [1,2,4,10] were as follows: lateral distal femoral angles (LDFA), medial proximal tibial angles (MPTA), proximal and distal epiphyseal and physeal gaps in the tibia and fibula, fibula and tibia length, fibula/tibia length ratio, tibiotalar angles, locations of osteochondromas in the tibia or fibula, and the presence of tibiofibular synostosis.

**Figure 1:**
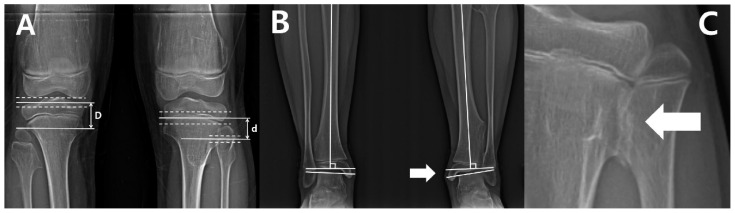
(**A**) The distance of proximal epiphyses (D) and physes (d) between tibia and fibula. The distance of proximal epiphyses was defined as the gap between the parallel line past the apex of the proximal fibula and the parallel line past the center of the distance between the line across the apex of the tibial spine and the most bottom of the condyle. The distances of proximal physes was defined as the gap between the parallel line passing through the midpoint of the distance between the line across the top of physis; the line crossing the bottom of physis was set as the reference line. (**B**) The measurement of the ankle joint. The tibiotalar angle was defined as the extension line of the tibial anatomical axis and a line perpendicular to the extension line that touches the dome of the talus. The left lower extremity indicated by the white arrow demonstrates a decreased distal tibiofibular distance compared to the right side, and a prominent ankle valgus deformity is also observed. (**C**) The tibiofibular synostosis. The white arrow indicates a definite bridge of an exostosis lesion connecting the proximal fibula and tibia without the overlapping cortex.

Anatomical LDFA was defined as the lateral angle between the longitudinal axis of the femoral shaft and a line across the surface of the distal femoral epiphysis. Mechanical MPTA was defined as the medial angle between the longitudinal axis of the tibial shaft and a line across the tibial plateaus. The fibular length was measured as the straight-line distance from the tip of the proximal epiphysis to the tip of the lateral malleolus; the tibial length was measured as the straight-line distance from the tips of the tibial eminences to the tibial plafond. We also measured the distance between the tips of the tibial and fibular epiphyses, respectively. Proximally, due to the complex anatomy of the proximal tibial epiphysis, the distance was defined as the gap between a parallel line past the apex of the proximal fibula and a parallel line through the midpoint between the line along the apex of the tibial spine and a line on the bottom-most portion of the condyle. Additionally, a parallel line through the midpoints between a line passing the top of the physis and a line crossing the bottom of physis were set as a reference line in order to measure the distance between the proximal and distal fibular and tibial physes (Figure 1A). The tibiotalar angle was defined as the medial angle between a perpendicular line to the axis of the tibia and the extended line that touches the articular surface of the talus (Figure 1B) [6]. In order to categorize the initial ankle alignment, the cases were divided into three positions: neutral (tibiotalar angle = 0–5°), valgus (tibiotalar angle > 5°), and varus (tibiotalar angle < 0°) [13].

The presence of tibiofibular synostosis was determined when definite bridging by an exostosis lesion of the tibia and fibula, without overlapping cortices, was identified (Figure 1C). The location of osteochondromas were determined by dividing the entire length of the fibula and tibia, including both ends of the proximal and distal epiphysis, into two equal parts [10].

Reliability testing of radiographic measurements was performed. Two orthopedic surgeons independently measured the radiographic parameters, and inter-observer reliability of the two surgeons was determined using the intraclass correlation coefficient (ICC). Both surgeons also repeated measurements of the same subject at 2-week intervals to assure intra-observer reliability.

### 2.3. Statistical Analyses

The intra- and inter-observer reliabilities of the radiographic measurements were analyzed using the ICC. ICC was calculated using a two-way mixed effect for an absolute agreement between both values of each observer’s measurements. An ICC value of 0.75 or greater was considered to reflect excellent reliability [14]. All statistical analyses were performed using SAS version 9.4 (SAS Institute, Cary, NC, USA) and R statistics 3.6.2. *p* value < 0.05 was indicated as statistically significant.

Numerical variables were presented as mean ± standard deviation and frequency (percentage). Independent two-sample t tests were used to compare patient demographics and radiographic measurements between the groups of N and V. Categorical variables such as sex, the presence of tibiofibular synostosis, and location of osteochondroma were compared using a chi-square test (Fisher’s exact test). Mann–Whitney U-test was used to compare the follow-up duration between the groups. The linear mixed model, for a repeated-measures random intercept model, was used for LDFA, MPTA, proximal and distal epiphyseal and physeal gaps between the tibia and fibula, tibia and fibula length, fibula/tibia length ratio, and tibiotalar angle. The changes in the fibular length, tibial length, fibula/tibia ratio, and tibiotalar angle according to time were considered as covariate x time interactions. The fixed effects of associated factors on tibial and fibular lengths, and the tibiotalar angle per month which can affect the growth of lower extremities including patient’s age, sex, synostosis and initial ankle alignment were analyzed.

## 3. Results

### 3.1. Inter-and Intra-Observer Reliability

The interobserver reliability for the radiographic measurements of the lower-limb alignment were found to be excellent (ICC; LDFA, 0.973; MPTA, 0.947; fibular length, 0.999; tibia; length, 0.999; proximal physis gap, 0.989; proximal epiphysis gap, 0.979; distal physis gap, 0.964; distal epiphysis, 0.953; tibiofibular angle, 0.993). The intraobserver reliability for the observer’s repeated measurements also resulted in excellent results (ICC; LDFA, 0.946; MPTA, 0.968; fibular length, 0.999; tibia; length, 0.999; proximal physis gap, 0.995; proximal epiphysis gap, 0.990; distal physis gap, 0.985; distal epiphysis, 0.968; tibiofibular angle, 0.999).

### 3.2. Demographic Distribution

The demographic data of patients at their first visit are summarized in Table 1.

Age at the initial visit did not differ between the groups; however, the follow-up period for group V was significantly longer than that of group N (group N, 27.1 ± 14.5 months; group V, 40.5 ± 19.8 months, *p* = 0.004). Group V also had a significantly greater number of radiographs taken for each leg than group N (*p* = 0.003). The distribution of initial ankle alignment in both groups was similar. Neutral alignment was most commonly seen, followed by valgus and varus alignments. There were no significant differences in the location of exostosis (proximal tibia, *p* = 0.250; distal tibia, *p* = 0.176; proximal fibula, *p* = 0.262, and distal fibula, *p* > 0.999, respectively) and the presence of tibiofibular synostosis (*p* = 0.906) between the groups. The mean tibiofibular alignments at the initial and last evaluation are displayed in Table 2. Based on the initial evaluation, no significant differences in any of the parameters were noted between the groups. However, the final evaluation revealed significant differences between the groups in all parameters, except for the distal physes gap.

### 3.3. Change in Alignment During the Observation Period

**Figure 2:**
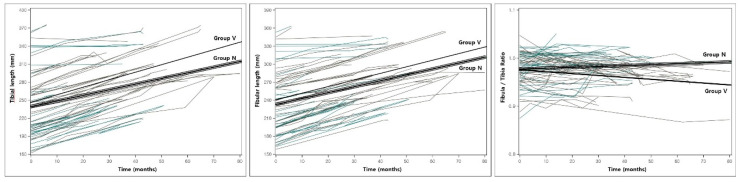
The spaghetti plot and estimated slope in the tibial, fibular length and fibula/tibia ratio of groups N and V. The solid line indicates the estimated slope of group V. and triple lines indicate the estimated slope of group N. No significant difference was noted in fibular length, and there was a significant difference in slope between group N and group V in tibial length and fibula/tibia ratio.

**Figure 3:**
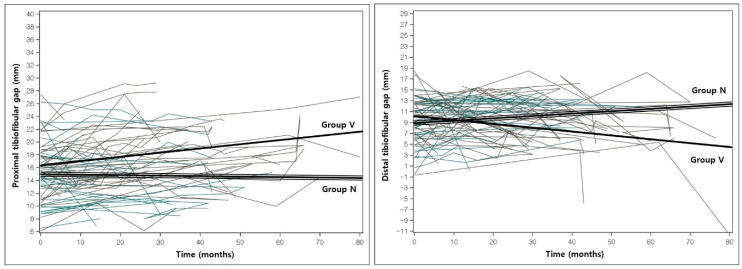
The spaghetti plot and estimated slopes in proximal and distal tibiofibular gaps of groups N and V. The solid line indicates the estimated slope of group V; and triple lines indicate the estimated slope of group N. Progressive fibular shortening with a significant increase in the proximal gap and a decrease in the distal tibiofibular gap were identified over time.

The estimated slopes of change between the initial and final measurements were analyzed using a linear mixed model. Group N had a significant constant increase in the proximal physes gap (0.062, *p* < 0.001) and the fibular (0.977, *p* < 0.001) and tibial (0.941; *p* < 0.001) lengths. Group V had a significant increase in the tibia and fibular length, the proximal physes and epiphyses gap, and the tibiotalar angle (Figure 2 and Figure 3), but a decrease in the LDFA, the fibula/tibia ratio, and the distal physes and epiphyses gap. In terms of the differences between the slopes of both groups, significant differences were identified in the tibial length, the fibular/tibial length ratio, the proximal and distal epiphyses gap, and the tibiotalar angle (Table 3).

### 3.4. Effects of Characteristic Factors

**Figure 4:**
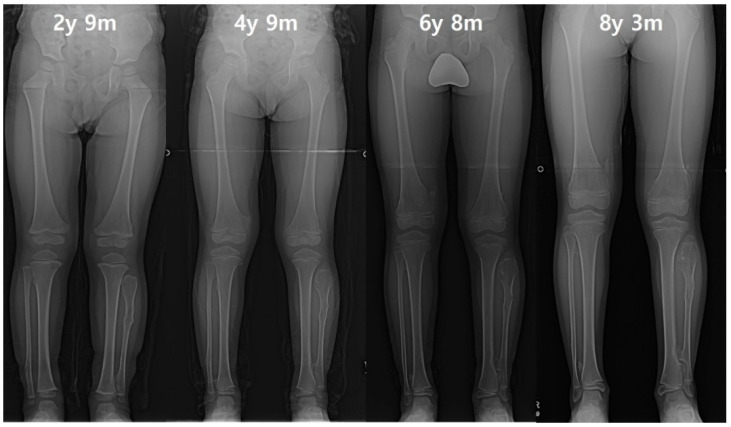
The serial radiographs of a male patient demonstrated progression of valgus deformity on the left ankle. Despite exostoses in bilateral legs, valgus angulation of the left ankle accompanied by shortening of the fibula was observed, while the neutral alignment was maintained on the right ankle. In the last measurement, the left tibial length was also about 9 mm shorter than the right tibia.

**Figure 5:**
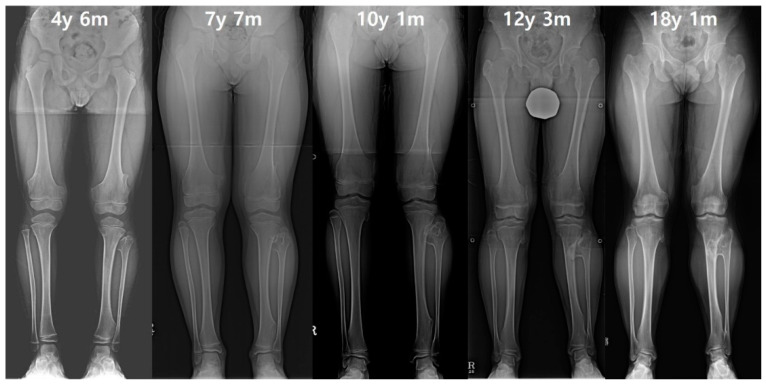
A male patient had prominent exostosis on the left proximal fibula and an increased gap of the proximal tibia and fibula at initial evaluation. Although the right lower extremity initially maintained a normal tibiofibular relationship, after the growth was completed, the right ankle joint developed valgus deformity to be equivalent to the left ankle. Finally, the leg length discrepancy was approximately 12 mm due to the 10 mm shortened length of the left tibia.

The fixed effects of the patient’s age, sex, synostosis and initial ankle alignment on subsequent tibial and fibular lengths, and the tibiotalar angle, are listed in Table 4. The amount of change per a month in the fibular and tibial lengths and the ratio of the fibular and tibial lengths decreased significantly (Figure 4). Sex differences were found to not affect the changes in the tibiofibular length ratio or tibiotalar angle. In the presence of synostosis, the rate of growth per month of the tibia and fibula decreased significantly (tibia, −0.125 mm/month; fibula, −0.138 mm/month); however, it did not affect the changes in the ratio of the fibular and tibia lengths or tibiotalar angle. When the initial ankle alignment was valgus, the ratio of the fibular and tibial length increased by 0.0005 per month (*p* = 0.004). For ankles with an initial varus alignment, significant increases in the tibiotalar angle were demonstrated (0.044 mm per month, *p* = 0.034) (Figure 5).

## 4. Discussion

In this study, we compared the longitudinal changes between the tibiofibular relationship and ankle alignment in HME patients. When the valgus angulation of the tibiotalar angle progressed, we found that the tibial growth was relatively greater than the fibular growth and was accompanied by significantly relative fibular shortening in the proximal and distal portions. This is in line with previous observations that have reported that HME of the lower extremities induces a disproportionate shortening of the fibula with respect to the tibia, leading to progressive valgus deformities of the ankle joint [10,11,15]. Additionally, the sex of the patient was identified to not affect the growth of the tibia or fibula, and synostoses was shown to slow the growth of both the tibia and fibula.

Although the absolute growth rates of the fibula cannot be directly compared, it may be assumed that the relative retardation of fibular growth caused the ankle valgus deformity and fibular shortening [4,13]. For children aged 7–10 years, during physiological growth, the proximal and distal tibiofibular physis differences are usually measured as 1.2 to 1.7 cm and 0.7 to 1.0 cm, respectively. However, in our study, the proximal and distal tibiofibular physis differences were greater than this, and, therefore, we assumed that the fibular growth retardation was apparent [13]. A similar phenomenon may also occur in the forearm, as deformities are caused by relative ulnar shortening [16]. The size of the osteochondromas in the ulna has been inversely correlated with the rate of ulnar shortening, and the location of osteochondromas on the distal ulnar metaphysis has been shown to restrict longitudinal bone growth to under 20% of their expected growth [17]. Using a longitudinal observation study design, we found that the fibula growth slopes were similar regardless of the direction of the change in the ankle alignment. However, for the tibia, significantly greater growth slopes were noted when ankle valgus angulation was present. Therefore, these changes in the tibiofibular relationship of HME patients support the effectiveness of surgical treatment of the tibia with procedures such as medial malleolar screw epiphysiodesis [8,9].

In studies investigating tibial and fibular growth in normal children, progressive distal migration of the distal fibular epiphysis and physis occurred with increasing age [3,13,18,19]. In cases of pediatric ankle fractures or iatrogenic tibiofibular synostosis, it has been reported that the tibiofibular synostosis blocks the physiological distal migration of the fibula, resulting in a proximal fibular migration and deformity with a prominent fibular head and shortened lateral malleolus [5,20,21,22,23]. However, our study revealed continuous shortening of both the proximal and distal fibula over time. In addition, the presence of synostosis was found to inhibit the growth of both the tibia and fibula when compared to the growth rates of those without synostoses. Therefore, these findings suggest that the fibular shortening seen in HME patients appears to show different trends with those observed by fractures or iatrogenic synostosis [21].

Takikawa et al. utilized a longitudinal study design with serial ankle radiographs and reported that an ankle valgus deformity could be predicted in male HME patients with prominent shortening of the fibula and exostoses involvement of both the lateral distal tibia and the medial distal fibula [11]. However, they evaluated a total of 62 ankle radiographs of 33 patients (23 males, 10 females) with a mean age of 11.33 years and did not conduct any statistical analysis related to the passage of time. In contrast, we found no significant fixed effects of sex on the growth of the tibia and fibula and no differences between sex or location of the exostosis on the ankle valgus deformity progression. No significant difference was found in age and sex between our groups, and it is possible that the difference in patient distributions compared to the study of Takikawa et al. may have led to the differing results.

A study evaluating the effect of osteochondroma locations on coronal lower limb malalignment reported that the combined lesions of both the proximal and distal tibiofibular joints had the greatest impact on fibular shortening [10]. In our study, the analysis of the combination lesion was not included, and no significance was found in the location of lesions in relation to fibular shortening, regardless of whether valgus progressed or not. Osteochondromas develop and grow while the physis is still open, and with variations in sex, they grow proportionately to the overall growth of the patient [19]. Therefore, bone age may influence the tibiofibular growth and relationship. Our study investigated the alignment changes over time within a cohort of patients all almost under the age of 10, and, hence, there may be differences between our results and those of the cross-sectional study of Ahn et al. on patients with a mean age of 10–14 years [10].

The initial ankle alignment did not significantly affect the future growth of the tibia and fibula in our study. Despite variations with age, minor valgus alignments of 0–8 degrees for the tibiotalar angle are within the standard range during skeletal development [13]. Noonan et al. investigated the natural history of 38 adult patients, mean age of 42 years, with lower extremity and ankle joint HMEs. Their mean tibiotalar angle was 8.6°, half of their cohort (50%) suffered from occasional ankle pain, and 14 of 75 ankles (19%) showed degenerative changes [6]. Although physiologic valgus alignment may be acceptable during the growing period, ankle valgus deformities that persists into adulthood can progress to arthritis and result in deterioration of the ankle joint function. More than 80% of patients with HME are initially diagnosed in the first decade of life. Therefore, careful observation and appropriately timed treatment during the growth period are required to minimize functional impairment and the progression of deformities [4,6].

In a retrospective review study of 113 HME patients, the distribution of osteochondromas was reported to be more likely in the proximal tibia (71%) than the distal tibia, and the case was similar for the proximal and distal fibula (27%) [24]. We also found that the location of osteochondromas occurred more proximally in both the tibia and fibula, a finding which is similar to those previous reported. Therefore, as osteochondromas may arise more proximally, evaluations of full length tibia and fibula radiographs may more reliably predict the progression of ankle valgus than those of ankle radiographs only.

This study has several limitations. First, the duration of follow-up was relatively short, and the follow-up time interval was irregular due to the retrospective study design. A prospective study with extended observations to adulthood and controlled time intervals may provide more reliable information to further our understanding of the progression of angular malalignment of lower extremities during the growth period for HME patients. Second, radiographic evaluation of patients was exclusively performed by teleoroentgenogram, with the center of the beam projection facing the knee. Therefore, there may be an inherent error with this beam projection set-up, and all values should have been compared with simultaneous ankle AP radiographs [25]. Nevertheless, since this study investigated the changes in the tibiofibular relationship over time, depending on the progression of ankle valgus deformity, we focused more on analyzing the overall trends of radiographic changes longitudinally. Last, the accuracy of the observations for the presence of synostosis and evaluation of osteochondroma sizes were insufficient due to the lack of a tomographic examinations. Tomographic scanning has the advantage of accurately demonstrating the condition, location, and synostosis of osteochondroma lesions [20]. However, it is clinically difficult to implement and justify such imaging in the pediatric HME population.

## 5. Conclusions

In pediatric HME patients, when valgus deformity of the ankle joint progressed, greater relative tibial growth and significantly relative proximal and distal fibular shortening followed. For the fixed effect of characteristic factors on the tibiofibular alignment over time, tibiofibular synostosis lowered the growth of both the tibia and fibula, whilst initial ankle alignment partially affected the tibiofibular relationship. However, demographic and anatomical factors such as age, sex, location and synostosis were not associated with the progression of ankle valgus deformities. Our observations suggest that pediatric HME patients with an imbalanced tibiofibular relationship, due to greater tibial growth, may require appropriate surgical treatment to prevent the progression of ankle valgus deformities, regardless of predisposing factors.

## Figures and Tables

**Table 1:** Summary of the patients.

	Group N (*n* = 28)	Group V (*n* = 35)	*p* Value
Numbers of affected leg (right:left)	15:13	18:17	
Age at first visit (months)	77.6 ± 40.8	82.8 ± 33.9	0.586
Sex (male:female)	14:14	17:18	0.910
Duration of follow-up (months)	27.1 ± 14.5	40.5 ± 19.8	0.004 *
Numbers of radiologic follow-up	3.4 ± 1.6	4.8 ± 2.1	0.003 *
Initial ankle alignment ^†^			0.964
Neutral (tibiotalar angle 0°–5°)	14 (51.9%)	17 (48.6%)	
Valgus (tibiotalar angle > 5°)	7 (26.0%)	10 (28.6%)	
Varus (tibiotalar angle < 0°)	6 (22.2%)	8 (22.9%)	
Location of osteochondroma			
Proximal tibia	17 (63.0%)	26 (74.3%)	0.250
Distal tibia	12 (44.4%)	21 (60.0%)	0.176
Proximal fibula	18 (66.7%)	27 (77.1%)	0.262
Distal fibula	12 (44.4%)	15 (42.9%)	>0.999
Tibiofibular synostosis			0.906
None	10 (37.0%)	12 (34.3%)	
Presence	18 (66.7%)	23 (65.7%)	

Values are presented as mean ± standard deviation or as numbers only. * Statistical significance was noted. ^†^ The valgus angle is expressed as a positive value, and the varus angle as a negative value.

**Table 2:** Measurements of lower limb alignment at initial visit and last follow-up.

	Group N	Group V	*p* Value
Initial visit			
Lateral distal femoral angle (°)	89.1 ± 4.2	87.7 ± 2.5	0.111
Medial proximal tibial angle (°)	89.3 ± 3.1	88.9 ± 2.3	0.567
Length of fibula (mm)	233.4 ± 59.0	240.8 ± 47.2	0.587
Length of tibia (mm)	239.9 ± 61.1	246.9 ± 48.4	0.620
Fibula/tibia ratio	0.97 ± 0.04	0.98 ± 0.34	0.834
Gap of proximal physis (mm)	11.9 ± 5.8	14.8 ± 6.0	0.058
Gap of proximal epiphysis (mm)	15.0 ± 5.0	15.9 ± 4.5	0.495
Gap of distal physis (mm)	6.7 ± 3.8	6.6 ± 3.6	0.905
Gap of distal epiphysis (mm)	8.6 ± 4.0	9.7 ± 4.3	0.329
Tibiotalar angle (°) ^†^	2.6 ± 7.0	2.9 ± 5.5	0.876
Last follow-up			
Lateral distal femoral angle (°)	88.4 ± 3.0	86.5 ± 2.3	0.007 *
Medial proximal tibial angle (°)	90.5 ± 3.3	88.1 ± 2.6	0.003 *
Length of fibula (mm)	260.9 ± 47.7	284.9 ± 42.0	0.040 *
Length of tibia (mm)	265.9 ± 50.2	296.9 ± 43.9	0.012 *
Fibula/tibia ratio	0.98 ± 0.03	0.96 ± 0.03	0.005 *
Gap of proximal physis (mm)	13.5 ± 5.8	19.2 ± 5.5	<0.001 *
Gap of proximal epiphysis (mm)	14.4 ± 4.9	19.1 ± 4.8	<0.001 *
Gap of distal physis (mm)	6.3 ± 3.6	5.2 ± 3.9	0.279
Gap of distal epiphysis (mm)	10.1 ± 3.7	6.0 ± 4.9	0.001 *
Tibiotalar angle (°) ^†^	1.6 ± 7.0	7.5 ± 6.2	0.001 *

Values are presented as mean ± standard deviation or as numbers only.* Statistical significance was noted. ^†^ The valgus angle is expressed as a positive value, and the varus angle as a negative value.

**Table 3:** Estimated slope of change between the initial and final measurements.

	Estimated Slope (SE)					
	Group N	*p* Value	Group V	*p* Value	Difference between Group N Versus. Group V	*p* Value
Lateral distal femoral angle (°)	−0.028 (0.0144)	0.052	−0.0266 (0.0081)	0.001 *	0.0015 (0.0165)	0.929
Medial proximal tibial angle (°)	0.0256 (0.0148)	0.087	−0.0026 (0.0084)	0.753	−0.0282 (0.017)	0.100
Length of fibula (mm)	0.9765 (0.0612)	<0.001 *	1.0968 (0.0344)	<0.001 *	0.1203 (0.0702)	0.088
Length of tibia (mm)	0.9407 (0.0622)	<0.001 *	1.2483 (0.035)	<0.001 *	0.3076 (0.0713)	<0.001 *
Fibula/tibia ratio	0.0002 (0.0001)	0.066	−0.0004 (0.0001)	<0.001 *	−0.0006 (0.0001)	<0.001 *
Gap of proximal physis (mm)	0.0623 (0.0166)	<0.001 *	0.0978 (0.0093)	<0.001 *	0.0354 (0.019)	0.064
Gap of proximal epiphysis (mm)	−0.0079 (0.0167)	0.637	0.0667 (0.0094)	<0.001 *	0.0746 (0.0192)	<0.001 *
Gap of distal physis (mm)	−0.0084 (0.0145)	0.563	−0.0177 (0.0081)	0.031 *	−0.0093 (0.0166)	0.574
Gap of distal epiphysis (mm)	0.0425 (0.0219)	0.054	−0.0702 (0.0124)	<0.001 *	−0.1126 (0.0251)	<0.001 *
Tibiotalar angle (°) ^†^	−0.0231 (0.0171)	0.180	0.0972 (0.0097)	<0.001 *	0.1202 (0.0197)	<0.001 *

Values are presented as mean (SE, standard error) or as numbers only. * Statistical significance was noted. ^†^ The valgus angle is expressed as a positive value, and the varus angle as a negative value.

**Table 4:** Interactive effects between the parameters at initial visit and the tibiofibular alignment.

	Fibular Length		Tibial Length		Fibula/Tibia Ratio		Tibiotalar Angle	
	Effect (SE)	*p* Value	Effect (SE)	*p* Value	Effect (SE)	*p* Value	Effect (SE)	*p* Value
Age	−0.005 (0.001)	<0.001 *	−0.004 (0.001)	<0.001 *	−0.000006 (0.0000018)	0.001 *	0.0001 (0.0003)	0.666
Sex								
Male	Ref. (0) ^†^		Ref. (0) ^†^		Ref. (0) ^†^		Ref. (0) ^†^	
Female	−0.108 (0.058)	0.062	−0.102 (0.063)	0.105	0.000003 (0.00012)	0.979	−0.017 (0.019)	0.360
Synostosis								
None	Ref. (0) ^†^		Ref. (0) ^†^		Ref. (0) ^†^		Ref. (0) ^†^	
Presence	−0.125 (0.058)	0.031 *	−0.138 (0.062)	0.028 *	0.0001 (0.0001)	0.492	−0.013 (0.019)	0.487
Initial ankle alignment								
Neutral (0°- valgus 5°)	Ref. (0) ^†^		Ref. (0) ^†^		Ref. (0) ^†^		Ref. (0) ^†^	
Valgus (> valgus 5°)	−0.032 (0.077)	0.673	−0.114 (0.083)	0.170	0.0005 (0.0002)	0.004 *	0.027 (0.025)	0.278
Varus (< 0°)	0.080 (0.063)	0.204	0.024 (0.068)	0.726	0.0002 (0.0001)	0.097	0.044 (0.021)	0.034 *

Values are presented as mean (SE, standard error) or as numbers only. Effects were analyzed using the linear mixed model and calculated as amount per month. * Statistical significance was noted. ^†^ The reference value is set to 0 to compare the effect of each factor among the parameters of tibiofibular alignment.
